# Genome‐wide association mapping of QTLs implied in potato virus Y population sizes in pepper: evidence for widespread resistance QTL pyramiding

**DOI:** 10.1111/mpp.12874

**Published:** 2019-10-11

**Authors:** Lucie Tamisier, Marion Szadkowski, Ghislaine Nemouchi, Véronique Lefebvre, Emmanuel Szadkowski, Renaud Duboscq, Sylvain Santoni, Gautier Sarah, Christopher Sauvage, Alain Palloix, Benoit Moury

**Affiliations:** ^1^ GAFL INRA 84140 Montfavet France; ^2^ Pathologie Végétale INRA 84140 Montfavet France; ^3^ UMR AGAP INRA F‐34060 Montpellier France; ^4^ Present address: Plant Pathology Laboratory TERRA‐Gembloux Agro‐Bio Tech University of Liège Passage des Déportés, 2 5030 Gembloux Belgium

**Keywords:** *Capsicum annuum*, effective population size, genome‐wide association, genotyping‐by‐sequencing, *Potato virus Y*, quantitative resistance, viral accumulation

## Abstract

In this study, we looked for genetic factors in the pepper (*Capsicum annuum*) germplasm that control the number of potato virus Y (PVY) particles entering the plant (i.e. effective population size at inoculation) and the PVY accumulation at the systemic level (i.e. census population size). Using genotyping‐by‐sequencing (GBS) in a core collection of 256 pepper accessions, we obtained 10 307 single nucleotide polymorphisms (SNPs) covering the whole genome. Genome‐wide association studies (GWAS) detected seven SNPs significantly associated with the virus population size at inoculation and/or systemic level on chromosomes 4, 6, 9 and 12. Two SNPs on chromosome 4 associated with both PVY population sizes map closely to the major resistance gene *pvr2* encoding the eukaryotic initiation factor 4E. No obvious candidates for resistance were identified in the confidence intervals for the other chromosomes. SNPs detected on chromosomes 6 and 12 colocalized with resistance quantitative trait loci (QTLs) previously identified with a biparental population. These results show the efficiency of GBS and GWAS in *C. annuum*, indicate highly consistent results between GWAS and classical QTL mapping, and suggest that resistance QTLs identified with a biparental population are representative of a much larger collection of pepper accessions. Moreover, the resistance alleles at these different loci were more frequently combined than expected by chance in the core collection, indicating widespread pyramiding of resistance QTLs and widespread combination of resistance QTLs and major effect genes. Such pyramiding may increase resistance efficiency and/or durability.

## Introduction

Cultivars carrying a major resistance gene have been extensively deployed to protect crops from pathogens. They have been widely used because they provide an almost complete resistance against pathogens, are environmentally friendly and can be easily introgressed by backcrossing. The major limit of this type of genetic control relies on the evolutionary potential of the pathogen population (García‐Arenal and McDonald, 2003; McDonald and Linde, 2002). Pathogen populations showing features such as high mutation rate, large effective population size and/or strong gene flow display high genetic diversity and pose the highest risk of evolution. At the same time, the widespread deployment of resistance genes in genetically uniform monocultures imposes a strong directional selection on the pathogen population, which can lead to the selection of better‐adapted pathogens and to the breakdown of the resistance gene. Several strategies have been proposed to control the pathogen evolution and limit its adaptation to the resistant plant, including pyramiding of several resistance genes or rotation of crops carrying different resistance genes (Mundt, 2002; Pink, 2002; Zhan *et al.*, 2015). Among these strategies, the combination of major resistance genes with quantitative resistance factors seems to be a promising alternative to ensure a durable crop protection. A greater durability of such polygenic resistance compared to monogenic resistance has been demonstrated in three pathosystems involving virus, fungus and nematode (Brun *et al.*, 2010; Fournet *et al.*, 2013; Palloix *et al.*, 2009). All these studies have observed a higher breakdown of the resistance gene when it was introgressed into a susceptible genetic background compared to a partially resistant one, probably because of the protective effect of the partially resistant genetic background on the major gene.

In the *Potato virus Y* (PVY; genus *Potyvirus*, family *Potyviridae*) – pepper (*Capsicum annuum*) system, the greater durability of the polygenic resistances is mostly due to the additional level of resistance conferred by the genetic background, which reduces viral accumulation (Quenouille *et al.*, 2013a). Smaller effective population sizes and greater genetic drift imposed by the genetic background were also probably involved in this higher resistance level. It means that, in small viral populations, the frequencies of the different viral strains within the population will randomly fluctuate over time independently of their fitness, allowing the loss of the most‐adapted virus variants by chance (Elena and Sanjuán, 2007). Several quantitative trait loci (QTLs) controlling virus accumulation or the effective population size have been previously identified using a pepper population of 153 doubled‐haploid (DH) lines issued from a crossing between two parental lines. All the DH lines were carrying the *pvr2* ^3^ major gene, which confers resistance against PVY, but were segregating for the genetic background. Two QTLs controlling PVY accumulation in the plant were detected and shown to be linked to the breakdown frequency of the major gene (Quenouille *et al.*, 2014). Three QTLs controlling the effective population size of the virus during the inoculation of the leaf were identified, one of them potentially colocalizing with a QTL on chromosome 6 controlling virus accumulation (Tamisier *et al.*, 2017). However, the confidence intervals of the two QTLs were large and barely overlapped, and it was therefore impossible to determine if the same genomic region was involved in both traits. Indeed, even if it is a powerful approach, QTL detection using a biparental population has two major limits. First, for each locus, the explored genetic diversity is low and corresponds to the polymorphisms distinguishing the parents. Secondly, the few recombination events in the population genealogy can limit the mapping resolution and lead to large QTL support intervals (Korte and Farlow, 2013). This is especially true for DH populations, which are the result of only one efficient meiosis. In this regard, genome‐wide association studies (GWAS) can be a complementary approach. Since GWAS are usually applied to large collections of theoretically unrelated individuals, the genetic diversity is supposed to be high and new alleles can be discovered. Furthermore, the high number of ancestral meioses that have occurred in the GWAS population can allow a precise QTL mapping (Hamblin *et al.*, 2011). For several plant species, the use of both GWAS and QTL mapping approaches have provided reliable results to dissect the genetic architecture of traits such as capsaicinoid content in pepper (Han *et al.*, 2018), plant agronomic features in soybean (Sonah *et al.*, 2015) or resistance to *Plum pox virus* (PPV) in *Arabidopsis thaliana* (Pagny *et al.*, 2012).

Recently, the availability of genotypes showing genetic backgrounds partially resistant to PVY in the pepper germplasm has been proved (Quenouille *et al.*, 2015). Among a collection of 20 pepper accessions, a high diversity of resistance levels conferred by the genetic background was observed, including for virus accumulation. In this context, the aim of the present study was to (i) select a core collection of pepper accessions and genotype them with a genotyping‐by‐sequencing (GBS) approach, (ii) perform GWAS for PVY effective population size at inoculation and PVY accumulation, and (iii) compare the results with those obtained by QTL mapping with a biparental DH population.

## Results

### A core collection representative of the pepper germplasm

Our goal was to select a core collection of *C. annuum* maximizing both the genetic and PVY resistance diversity. We first estimated the optimal size for the core collection by calculating the increase of allelic richness for different core collection sizes. The plateau was reached for 370 accessions but the allelic richness was very close for 310 accessions, with an allelic richness of 317 and 316, respectively (Fig. S1). We then applied the maximization strategy algorithm and obtained a core collection of 310 accessions that captured 91% of the alleles of the *C. annuum* collection (Table 1). The average number of alleles per locus in the core collection remains high relative to the whole collection (>12) as well as Nei’s unbiased gene diversity index (*H*_e_), which is constant (0.59). The observed heterozygosity remains unchanged and low (0.035), a result explained by the multiplication through selfing of the accessions and the preferential autogamy of *C. annuum*. Previously, the structure of the whole *Capsicum* spp. collection has revealed six distinct clusters (Nicolaï *et al.*, 2013). The cultivated *C. annuum* var. *annuum* accessions were distributed across the clusters 1, 2 and 3 while the wild *C. annuum* var. *glabriusculum* accessions were split between the cluster 1 and two additional clusters. The proportion of accessions belonging to each cluster was not significantly different between the whole and core collections (Fisher's exact test, *P* = 0.13, Table 1), attesting that the core collection sampled with the maximization strategy algorithm is an accurate representation of the *C. annuum* collection. After performing DNA extraction and GBS, 256 accessions were kept (Table S1).

**Table 1:** Comparison of the genetic diversity of the whole collection of *Capsicum annuum* and the core collection obtained with the maximization strategy algorithm. For each collection, the table provides the number of accessions, the percentage of SSR alleles represented in the collection, the mean number of alleles observed per locus, Nei’s unbiased gene diversity (*H*_e_), observed heterozygosity (*H*_o_) and the percentage of accessions belonging to each cluster defined by STRUCTURE v. 2.3.4 software based on SSR markers (Nicolaï *et al.*, 2012).

Sample	Sample size	% SSR alleles	Allele number	*H*_e_	*H*_o_	Distribution in the *C. annuum* clusters			
						Cluster 1	Cluster 2	Cluster 3	Clusters 4–6
Whole collection of *C. annuum*	887	100	12.57	0.59	0.035	36% (314)	21% (190)	41% (367)	2% (16)
Core collection of *C. annuum*	310	91	12.07	0.59	0.035	30% (93)	23.6% (73)	42.9% (133)	3.5% (11)

### Distribution of SNPs in the pepper genome

A total of 33 957 SNPs were first identified. After applying filters, 10 307 SNPs including 680 indels were kept (Table 2 and Fig. S2). An average sequencing depth of 140× was obtained. Among the SNPs, the transition/transversion (Ts/Tv) ratio was 1.46. A bias toward transition substitutions of the same order (1.35) has been previously observed in another study on 222 *C. annuum* genotypes (Taranto *et al.*, 2016). An average of 4 × 10^−6^ SNPs/bp was found in the entire genome, varying between 3.18 × 10^−6^ SNPs/bp on chromosome 10 and 6.01 × 10^−6^ SNPs/bp on chromosome 2 (Table 2). Finally, across the genome, linkage disequilibrium (LD) decayed on average after 8 kb, where *r* ^2^ = 0.2 (Fig. S3).

**Table 2:** Number and density of SNPs identified by ddRADseq in the pepper core collection.

Chromosome	Length (bp)	Number of SNPs	SNPs/bp
1	261 560 226	1027	3.93 × 10^−6^
2	166 118 313	998	6.01 × 10^−6^
3	241 745 451	1279	5.29 × 10^−6^
4	206 470 299	722	3.50 × 10^−6^
5	223 151 943	731	3.28 × 10^−6^
6	217 864 955	960	4.41 × 10^−6^
7	227 551 634	839	3.69 × 10^−6^
8	134 909 690	587	4.35 × 10^−6^
9	247 983 219	850	3.43 × 10^−6^
10	227 301 773	723	3.18 × 10^−6^
11	246 428 986	807	3.27 × 10^−6^
12	232 591 935	785	3.38 × 10^−6^

### Population structure and kinship relationships

**Figure 1:**
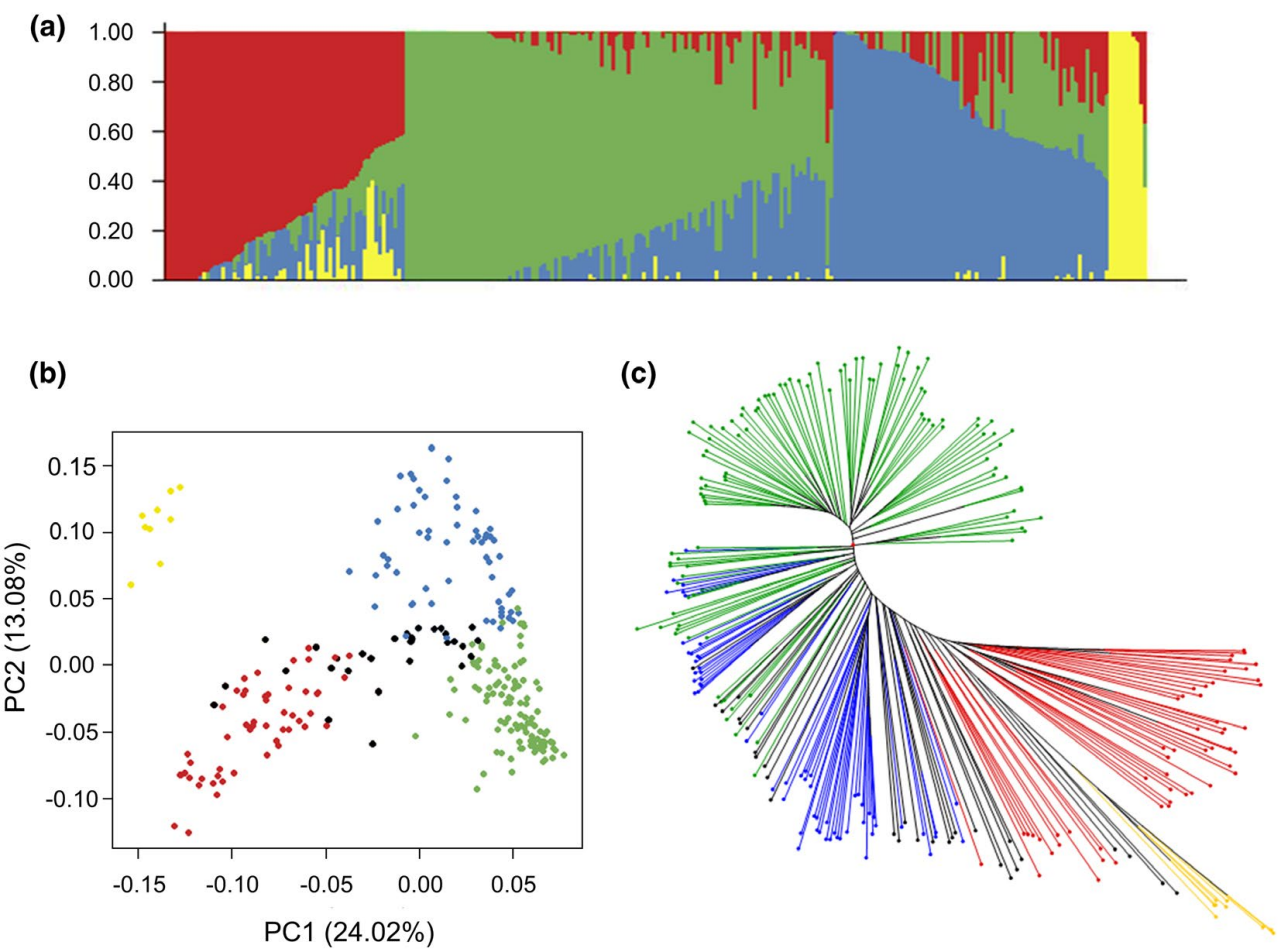
Population structure and genetic diversity of the *Capsicum annuum* germplasm core collection (256 accessions) on the basis of SNPs. (a) Classification of the core collection using STRUCTURE v. 2.3.4 software. Each vertical bar represents one pepper accession. (b) Principal component analysis of the core collection. (c) Neighbour‐joining phylogenetic tree of the core collection. For the three plots the colours green, blue, red and yellow stand for the four clusters defined by STRUCTURE software. For the last two plots, the accessions in black display strong admixture (membership coefficient <50% in each group).

Population structure analysis revealed that the pepper accessions were divided into four genetic clusters (*K* = 4), with some degree of admixture between the clusters (Figs 1a and S4). Principal component analysis (PCA) confirmed this result and also displayed four groups within the core collection (Fig. 1b). A neighbour‐joining tree based on pairwise genetic distances between SNPs was constructed. The clusters were clearly separated, supporting the previous results (Fig. 1c).

The first three genetic clusters (green, blue and red) included all the cultivated subspecies *C. annuum* var. *annuum*, while the fourth (yellow) was composed of the wild subspecies *C. annuum* var. *glabriusculum* only (Fig. 1). The genetic structure of the four clusters was strongly correlated to the morphological and developmental phenotypes of the accessions. The clusters differed significantly for several plant features, such as the fruit shape and length, the fruit pericarp thickness, the flowering date or the number of leaves (Fig. S5). The first cluster was mainly composed of sweet and large‐fruited peppers, the second cluster was composed of triangular and/or elongated fruits, and the third cluster included small and elongated fruits. These differences result from the long‐term selection imposed by farmers in multiple environments for quality traits but also for resistance to biotic and abiotic stresses. Finally, the fourth cluster showed very tiny ovoid fruits, a distinctive feature of the wild accessions belonging to this group.

The pairwise kinship matrix among the 256 individuals was assessed using the VanRaden equation (2008) as implemented in the Genomic Association and Prediction Integrated Tool (GAPIT) (Lipka *et al.*, 2012). The accessions showed a low level of relatedness (mean = 0.076). Fifty‐four percent of the accessions showed no relatedness (estimate of 0) and only 1.8% of pairwise kinship coefficients were higher than 0.5, indicating that most of the accessions are unrelated (Fig. S6).

### Variation in the number of primary infection foci and the virus accumulation among the pepper core collection

**Figure 2:**
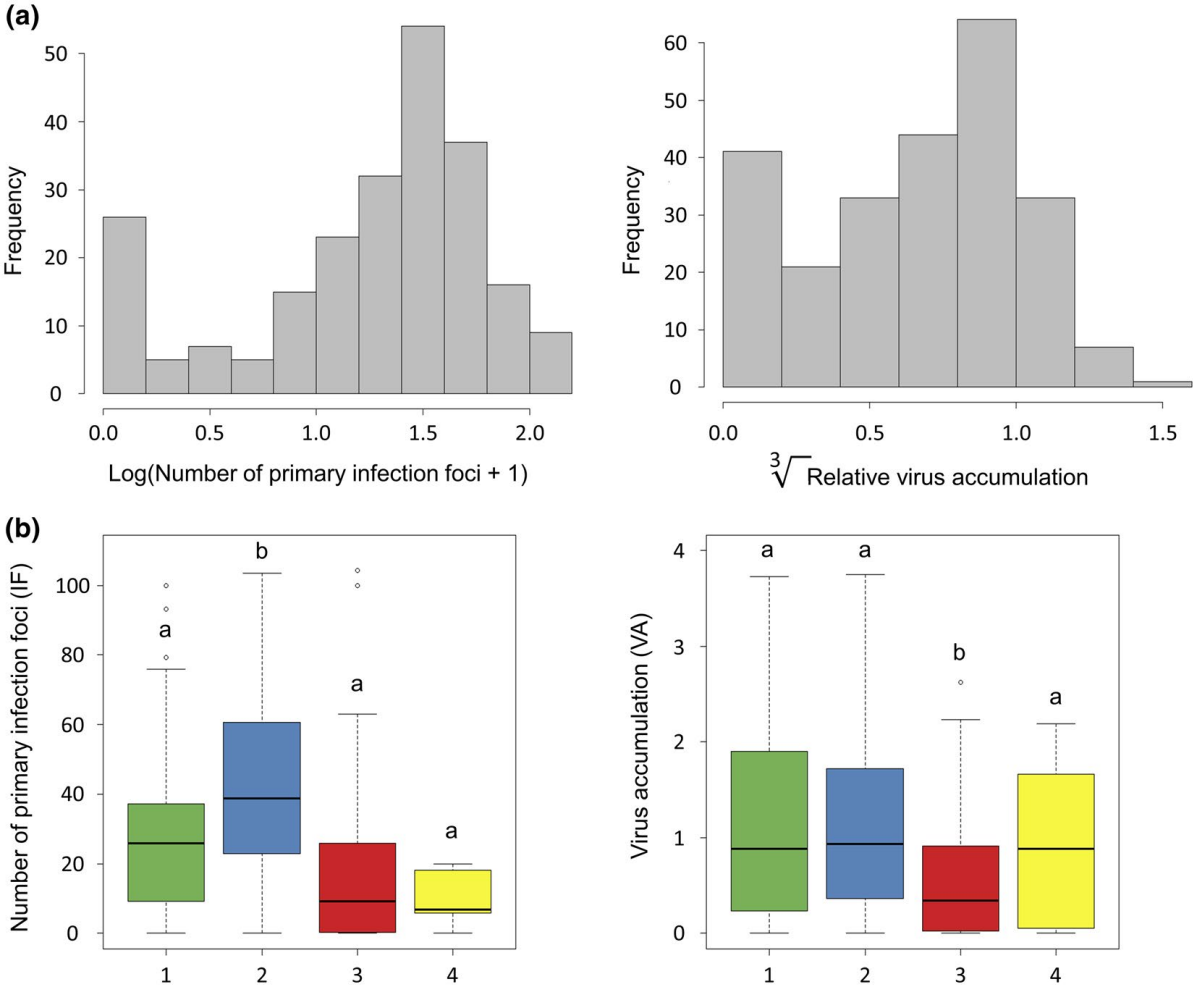
Potato virus Y resistance in the pepper core collection. (a) Frequency distribution of the number of primary infection foci (IF) caused by PVY‐GFP and of the relative virus accumulation (VA) in the *Capsicum annuum* core collection. (b) Distribution of IF and VA among the four clusters determined by the STRUCTURE analysis. The letters a and b indicate the different groups obtained after pairwise comparisons using the Nemenyi test (*P* < 0.05).

Two traits were phenotyped among the core collection: the number of primary infection foci (variable IF) induced by a green fluorescent protein (GFP)‐tagged PVY clone on the cotyledons, and the relative virus accumulation (variable VA) of a variant of PVY isolate SON41p in the apical leaves. Both traits were highly variable and showed significant differences between the accessions (*P* < 0.001, Kruskal–Wallis test), attesting to the large diversity of quantitative resistance in the pepper germplasm (Fig. 2a). The two traits showed a weak but significant positive correlation (Spearman ρ = 0.46, *P* < 0.001). They also both showed a high broad‐sense heritability, with *h* ^2^ = 0.98 for IF and *h* ^2^ = 0.80 for VA. The four genetic clusters previously identified were significantly contrasted for IF and VA (Fig. 2b). The accessions belonging to the second cluster had higher IF than the others on average, while the accessions of the third cluster showed a lower VA on average.

### Genome‐wide association mapping of pepper resistance to PVY‐SON41p variants

**Figure 3:**
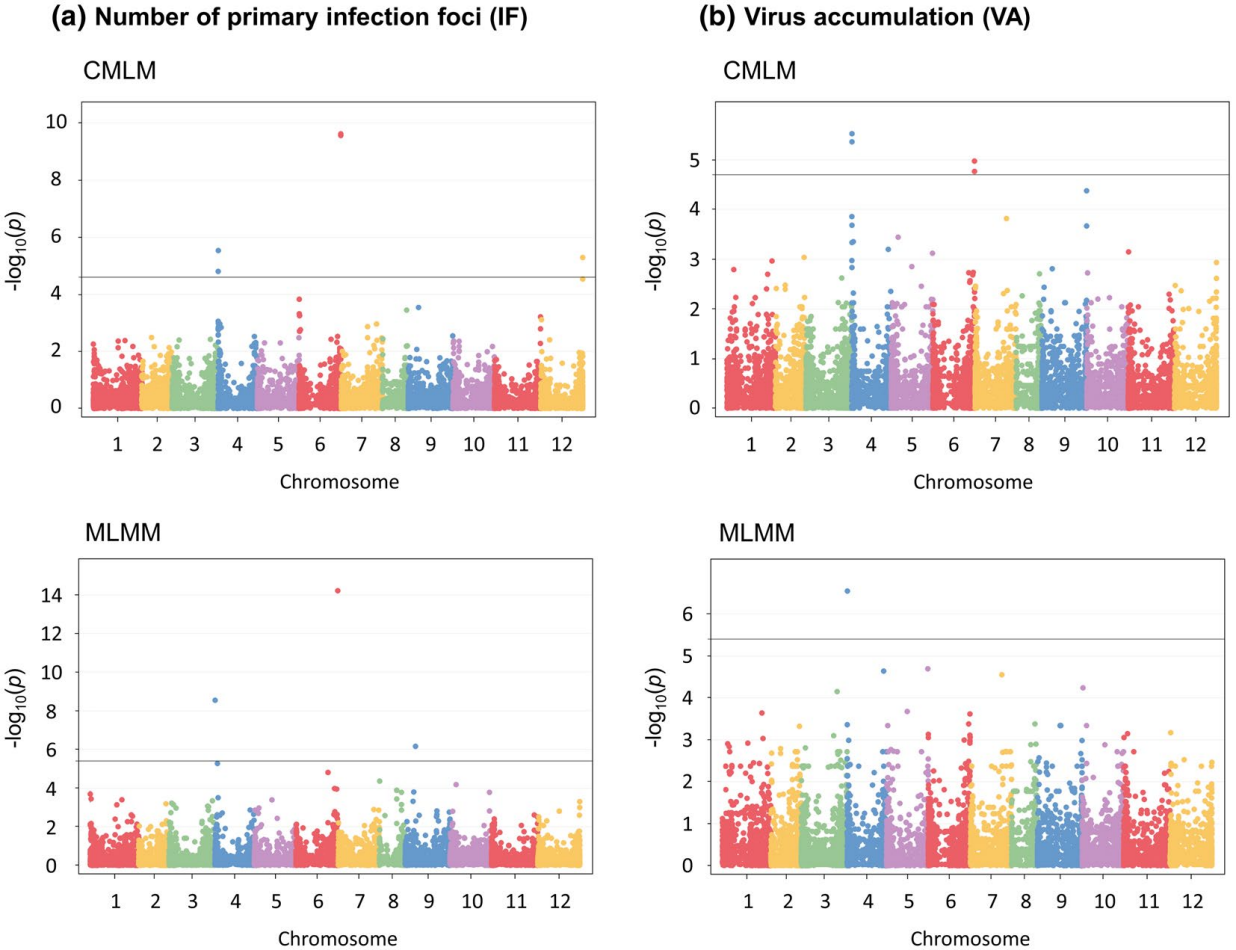
Manhattan plot of genome‐wide association studies (compressed mixed linear model, CMLM and multilocus mixed‐model, MLMM) for (a) the number of primary infection foci (IF) induced by PVY‐GFP and (b) PVY accumulation (VA). Negative log_10_(*p*) from a genome‐wide scan are plotted against SNP positions on each of the 12 chromosomes.

**Figure 4:**
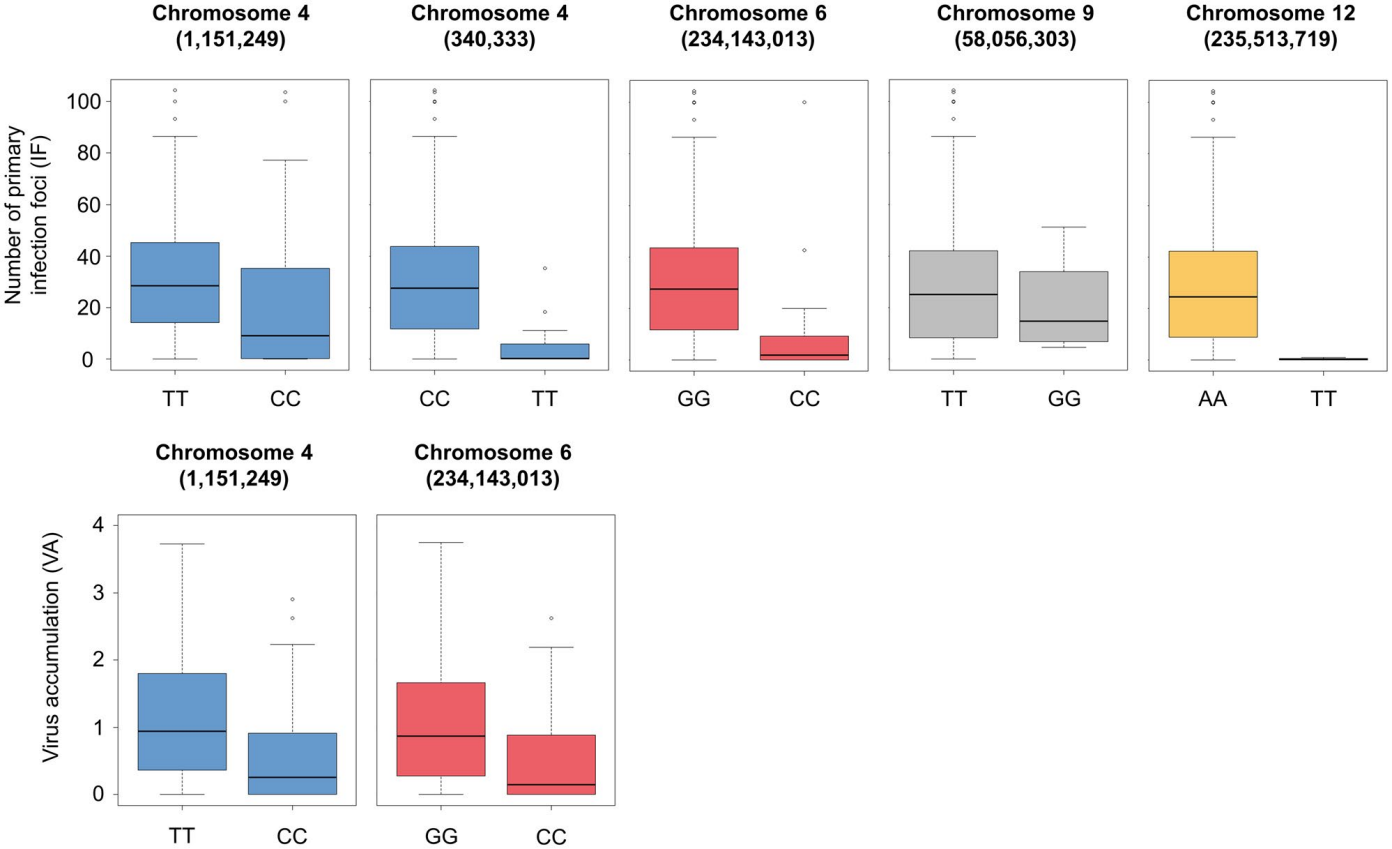
Allelic effect of the most significant SNPs detected with compressed mixed linear model (CMLM) and multilocus mixed‐model (MLMM) for the number of primary infection foci (IF) and PVY accumulation (VA). The position of the SNP on the CM334 pepper reference genome is indicated in parentheses (in bp).

A total of six SNPs localized on chromosomes 4, 6, 9 and 12 were significantly associated with IF (false discovery rate [FDR] and Bonferroni‐corrected threshold < 0.05 for compressed mixed linear model [CMLM] and multilocus mixed‐model [MLMM], respectively) (Table 3 and Fig. 3). Four SNPs localized on chromosomes 4 and 6 showed significant associations with VA. Among these markers, two SNPs on chromosome 6 and one SNP on chromosome 4 were detected for both traits with the CMLM. For the most significant SNP of each chromosome, the allelic effects on both traits were estimated (Fig. 4). All these SNPs were also detected when excluding the wild subspecies *C. annuum* var. *glabriusculum* from the analysis (Table S2).

**Table 3:** SNPs identified with genome‐wide association studies and associated with the number of primary infection foci (IF) induced by PVY‐GFP and PVY accumulation (VA) in pepper.

Trait	Chromosome number	Position (bp)	CMLM		MLMM	
			−log_10_(*p*)	*R* ^2^(%)	−log_10_(*p*)	*R* ^2^(%)
IF	4	340 333	4.80	6.8	8.55	13.5
	4	1 151 249	5.53	8.1	NS	NS
	6	234 143 013	9.62	15.4	14.21	13.9
	6	234 142 995	9.56	15.3	NS	NS
	9	58 056 303	NS	NS	6.16	7.8
	12	235 513 719	5.29	7.7	NS	NS
VA	4	1 151 249	5.53	8.0	6.55	11.0
	4	1 151 254	5.36	7.7	NS	NS
	6	234 143 013	4.97	7.1	NS	NS
	6	234 142 995	4.77	6.8	NS	NS

CMLM, compressed mixed linear model; MLMM, multilocus mixed‐model; NS, not statistically significant.

**Figure 5:**
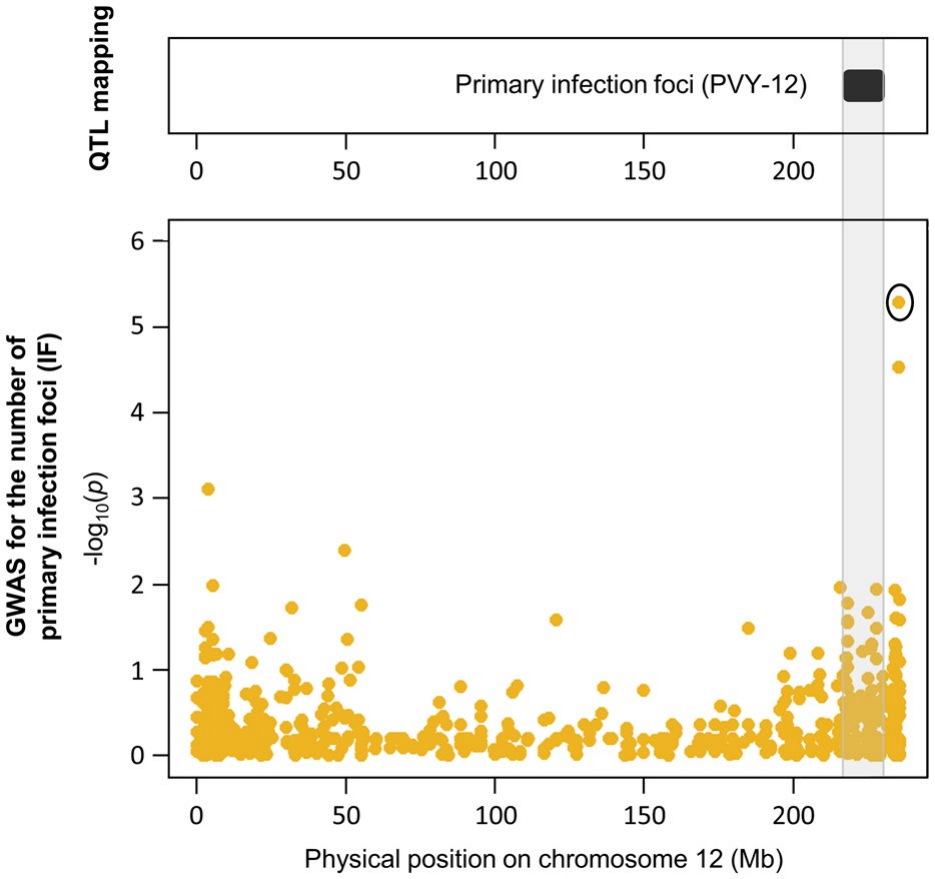
Genomic regions controlling the number of primary infection foci (IF) on chromosome 12. The PVY‐12 (black) quantitative trait locus (QTL) detected by analysis of a biparental DH progeny in Tamisier *et al*. (2017) is mapped physically on pepper chromosome (top box). SNPs are represented in Manhattan plots displaying the –log_10_(*p*) over genomic positions (bottom boxes). The SNP showing significant association with the trait is surrounded by a black circle. The shaded grey area delimits the boundaries of the PVY‐12 QTL.

**Figure 6:**
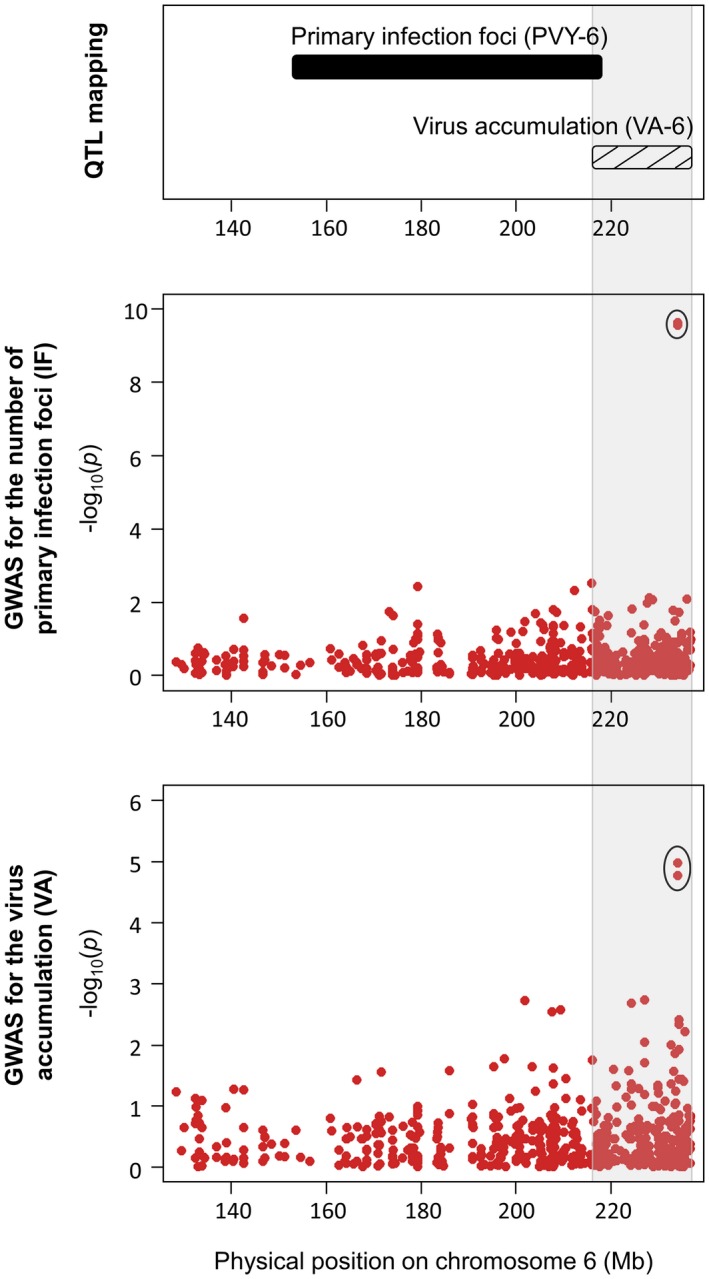
Genomic regions controlling the number of primary infection foci (IF) and virus accumulation (VA) at the bottom of chromosome 6. The PVY‐6 (black) and VA‐6 (hatched) quantitative trait loci (QTLs) detected by the analysis of a biparental DH progeny in Tamisier *et al*. (2017) and Quenouille *et al*. (2014) are mapped physically on pepper chromosome (top box). For both traits, SNPs are represented in Manhattan plots displaying the –log_10_(*p*) over genomic positions (bottom boxes). SNPs showing significant association with the traits are surrounded by a black circle. The shaded grey area delimits the boundaries of the VA‐6 QTL.

**Figure 7:**
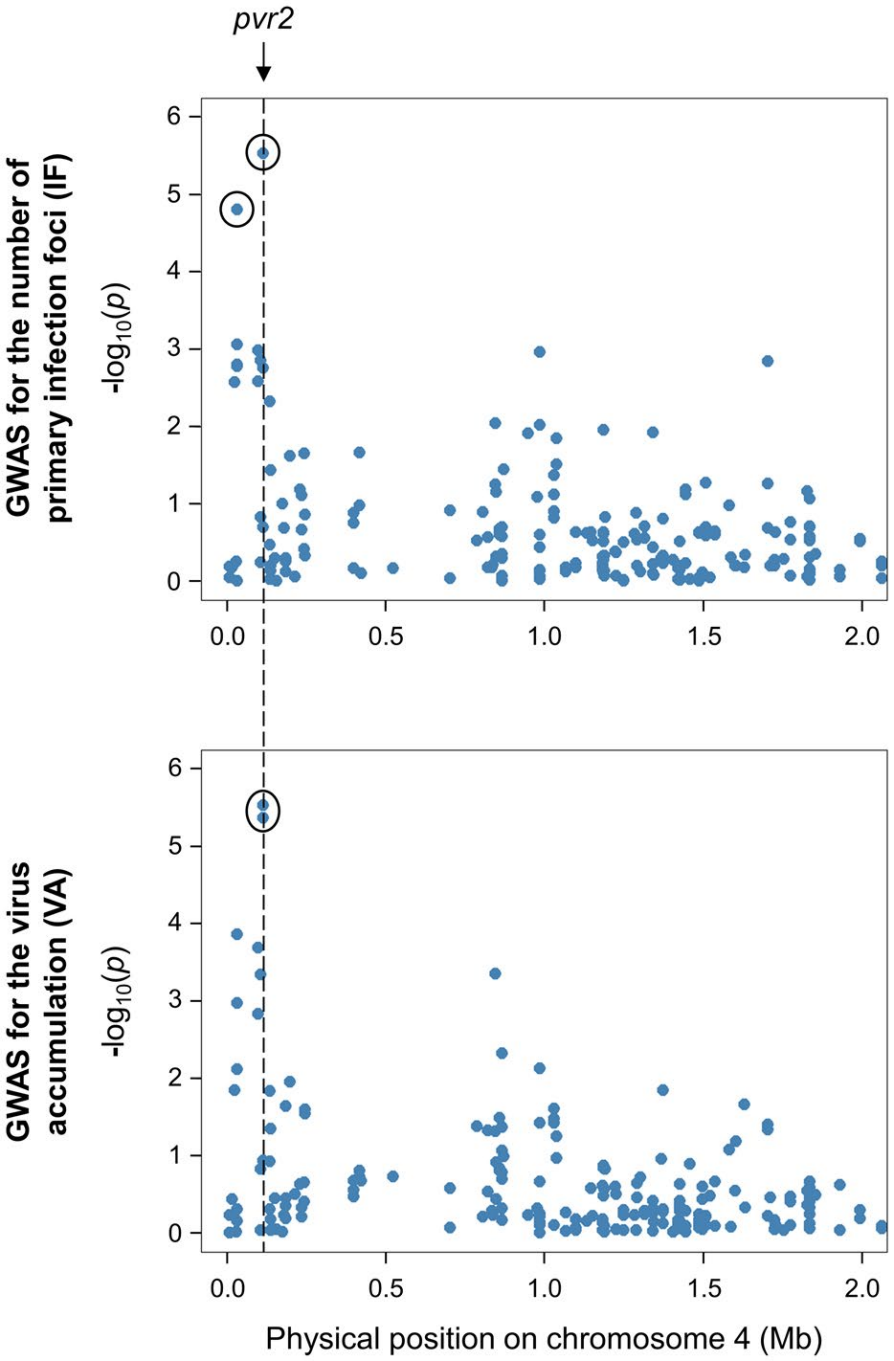
Comparison between SNPs mapped at the beginning of chromosome 4 (represented in a Manhattan plot displaying the –log_10_(*p*) over genomic positions) and the position of the major *pvr2* resistance gene on the CM334 reference genome (indicated with a dotted line). The SNPs showing significant association with the number of primary infection foci (IF) and/or virus accumulation (VA) are surrounded by a black circle.

These results were then compared with those previously obtained by QTL mapping with the biparental DH progeny for the same traits. For IF, three QTLs have been previously detected on chromosomes 6 (PVY‐6), 7 (PVY‐7) and 12 (PVY‐12) (Tamisier *et al.*, 2017). No significant association was found on chromosome 7 with GWAS. The SNPs detected on chromosomes 12 and 6 were approximately 10 Mb and 20 Mb distant from the QTLs PVY‐12 and PVY‐6, respectively (Figs 5 and 6). Regarding VA, two QTLs have been previously identified: one QTL on chromosome 3 (VA‐3), which colocalized with the *pvr6* gene, and one QTL on chromosome 6 (VA‐6) (Quenouille *et al.*, 2014). No significant association was found on chromosome 3 with GWAS. On chromosome 6, the confidence interval of VA‐6 lies between the physical positions 214 459 454 and 235 745 825 bp of the CM334 reference genome. Both SNPs associated with VA on this chromosome are included within this region (Fig. 6). Finally, no QTL has been previously identified on chromosomes 4 and 9. However, the *pvr2* locus is mapped between positions 1 193 194 and 1 197 686 bp on chromosome 4. The SNP detected for both traits on chromosome 4 is localized at position 1 151 249 bp, which is the closest marker linked to the *pvr2* gene among all the SNPs (Fig. 7).

### Significant associations between resistance alleles to PVY‐SON41p variants among the core collection

We analysed the distribution of SNPs detected in GWAS among the pepper core collection. The analysis was performed on the most significant SNPs identified on chromosomes 4 (positions 1 151 249 and 340 333 bp), 6 (position 234 143 013 bp), 9 (position 58 056 303 bp) and 12 (position 235 513 719 bp). Among the core collection, none of the accessions carried a resistance allele at the SNPs localized on the four different chromosomes at the same time, 11 accessions carried a resistance allele at the SNPs localized on three different chromosomes, and 30 accessions carried at least a resistance allele at two SNPs localized on two different chromosomes (Table S3).

We then analysed the distribution of resistance and susceptibility alleles for pairs of SNPs belonging to different chromosomes (Fig. S7). The resistance alleles of the SNPs on chromosomes 4 and 6 are carried together (i.e. are in coupling) more often than would be expected by chance (χ^2^ = 28.58, *P* = 3.7 × 10^−7^ for chromosome 4 position 1 151 249 bp and chromosome 6 position 234 143 013 bp detected with CMLM; χ^2^ = 6.44, *P* = 0.02 for chromosome 4 position 340 333 bp and chromosome 6 position 234 143 013 bp detected with MLMM). The same result has been found for SNPs on chromosomes 4 and 9 (χ^2^ = 25.53, *P* = 1.9 × 10^−4^ for chromosome 4 position 340 333 bp and chromosome 9 position 58 056 303 bp detected with MLMM), for SNPs on chromosomes 4 and 12 (χ^2^ = 5.89, *P* = 0.038 for chromosome 4 position 1 151 249 bp and chromosome 12 position 235 513 719 bp detected with CMLM), for SNPs on chromosomes 6 and 9 (χ^2^ = 40.75, *P* = 1.4 × 10^−6^ for chromosome 6 position 234 143 013 bp and chromosome 9 position 58 056 303 bp detected with MLMM) and for SNPs on chromosomes 6 and 12 (χ^2^ = 16.09, *P* = 0.005 for chromosome 6 position 234 143 013 bp and chromosome 12 position 235 513 719 bp detected with CMLM).

Finally, the mean interchromosomal LD estimates has been compared to the interchromosomal LD of the associated SNPs. The LD between SNPs on chromosomes 4 and 6 was *r* ^2^ = 0.02 for the SNPs detected with the MLMM and 0.11 for the SNPs detected with the CMLM (Fig. S8). These values were higher than the 60th and 80th percentiles of the LD interchromosomal distribution, respectively. The LD was 0.10 for the SNPs on chromosomes 4 and 9 (>85th percentile), 0.013 for the SNPs on chromosomes 4 and 12 (>55th percentile), 0.15 for the SNPs on chromosomes 6 and 9 (>90th percentile) and 0.05 for the SNPs on chromosomes 6 and 12 (>70th percentile).

## Discussion

### Benefits and limits of the pepper core collection to perform genome‐wide association

The aim of the present study was to estimate the availability and diversity of genetic factors controlling PVY population sizes in pepper genetic resources. Our strategy was to select a core collection composed of 256 pepper inbred lines representative of the genetic diversity of the pepper germplasm. The core collection has then been phenotyped for two traits linked to quantitative resistance to PVY‐SON41p variants, genotyped using GBS and association mapping has been performed.

Among the pepper accessions, the two traits phenotyped were IF, the number of PVY infection foci (isolate SON41p, mutant 115K) on inoculated pepper cotyledons, and VA, the PVY accumulation (isolate SON41p, mutant 119N) at the systemic level. IF is a proxy of the effective population size (*N*_e_), while VA is a proxy of the census population size. *N*_e_ can be defined as the number of viral particles that pass their genes to the next generation (Elena and Sanjuán, 2007). It is a parameter of interest when studying plant resistance durability because it is directly related to the evolution of the viral population. Indeed, the strength of genetic drift within the plant is linked to *N*_e_: when *N*_e_ is small, genetic drift is strong and virus variants can be lost independently of their fitness; when *N*_e_ is large, genetic drift is weak and the most‐adapted variants will increase in frequency within the plant because of the action of selection. Previous studies have demonstrated that during the inoculation of virus on the leaf, a primary IF is induced by only one viral particle (Tamisier *et al.*, 2017; Zwart *et al*., 2011). Therefore, quantifying the number of primary IF on a leaf is a direct estimation of *N*_e_ at inoculation. In contrast to IF, VA is related to the total number of individuals in the population. Therefore, it is an accurate estimator of the quantitative resistance conferred by the plant: the lower VA, the higher the quantitative resistance. IF and VA were highly variable among the pepper accessions (Fig. 2) and showed a high broad‐sense heritability, as previously reported for these traits on a DH pepper population (Quenouille *et al.*, 2014; Tamisier *et al.*, 2017). Hence, phenotype data were optimal to perform GWAS on both traits.

Model‐based analysis has structured the core collection within four clusters closely linked to plant features (Fig. 1). This observation was already reported in previous studies on *C. annuum* collections genotyped with SSR (Nicolaï *et al.*, 2013) or GBS data (Taranto *et al.*, 2016). Regarding the linkage disequilibrium, we found a rapid LD decay in the 256 accessions (8 kb at *r* ^2^ = 0.2). Taranto *et al. *(2016) also observed a rapid decay of LD (100 kb at *r* ^2^ = 0.2) in a collection of 222 *C. annuum* accessions. Multiple factors can cause changes in LD, such as bottlenecks, selective breeding or mating system (Flint‐Garcia *et al.*, 2003). The composition of the population can also greatly influence LD. In maize, Ersoz *et al. *(2007) reported that LD decays within 1 kb in land races (Tenaillon *et al.*, 2001), in approximately 2 kb in diverse inbred lines (Remington *et al.*, 2001) and can go up to 100 kb in commercial elite inbred lines (Ching *et al.*, 2002).

A rapid LD decay can be limiting in association mapping because it requires a high number of markers to cover the entire genome. Nevertheless, if the marker density is high enough, it can also be advantageous because the association signal will be closer to the causal polymorphism, leading to a high mapping resolution and consequently facilitate the introgression of the favourable alleles by backcross strategy (Flint‐Garcia *et al.*, 2003; Hamblin *et al.*, 2011). In our study, the rapid LD decay can also make the comparison between classical QTL mapping and GWAS difficult. Indeed, one way to compare the results of both studies is to map the QTLs on the reference genome and to see if the confidence intervals overlap between QTL and SNPs detected in association. Therefore, it is necessary to calculate a confidence interval around the associated markers detected in GWAS. There is no widely used method for this (Hayes, 2013), and the methods proposed need a large LD block around the marker in association to draw boundaries. This is usually not the case when LD is low, which generally leads to the detection of one or two significant SNPs without a large LD block around them (Cormier *et al.*, 2014). That was the case in our study, and we have therefore simply observed if the positions of the significant SNPs were close to or included in the support interval of previously detected QTLs. To estimate confidence intervals containing candidate genes, we defined upper and lower boundaries as the closest non‐detected SNP that had a similar major/minor allele frequency ratio as the one of the significant SNP. The rationale is that if this SNP (or SNPs beyond this boundary) were responsible for the observed phenotypic difference, it should have been detected by GWAS since there is no lack of power due to a greater major/minor allele frequency imbalance. Using this method, a total of 101 candidate genes that could be involved in IF and VA was obtained. The list of the candidate genes and a discussion about the best candidates are provided in the supporting information (Text S1, Table S4).

### Common genetic factors control PVY‐SON41p effective population size at inoculation and PVY‐SON41p accumulation

The association mapping has revealed seven different SNPs in association with IF and/or VA (Table 3 and Fig. 3). On chromosome 4, a common SNP was associated with both traits and the favourable allele was the same. All significant SNPs were close to the position of the *pvr2* resistance gene, which encodes a eukaryotic initiation factor 4E (eIF4E) and *pvr2* was included in the confidence interval of candidate genes (Fig. 7 and Table S4). To confirm that *pvr2* could be responsible for PVY‐SON41p resistance differences in the pepper core collection, we sequenced the corresponding open reading frame of 50 accessions randomly chosen among the resistant or susceptible phenotypic classes (Table S1). The association between the number of PVY IF and *pvr2* alleles was more significant than that observed between this phenotype and detected SNPs, confirming the probable involvement of the *pvr2* gene (see Text S1 for details).

On chromosome 6, the same associated SNPs were identified for IF (i.e. *N*_e_ at inoculation) and VA (Table 3 and Fig. 6). Furthermore, the favourable allele was the same for both traits (Fig. 4). These results suggest strongly than one or several gene(s) have a pleiotropic effect and control both *N*_e_ at inoculation and VA. Several studies have demonstrated that the number of founders entering the plant and the VA within the plant were related. Rodrigo *et al. *(2014) demonstrated that diminishing *N*_e_ at inoculation induced a delay in plant infection at the systemic level and Lafforgue *et al. *(2012) showed that it also resulted in a lower proportion of infected cells at the systemic level. Therefore, both mechanisms probably lead to a lower VA in the end. These SNPs lie within the support interval of VA‐6, a previously detected QTL controlling VA (Fig. 6). Finally, the SNPs associated with *N*_e_ at inoculation were at least 20 Mb apart from the support interval of the previously detected QTL PVY‐6 controlling the same trait. Therefore, one or two different genomic regions on chromosome 6 could control *N*_e_ at inoculation.

On chromosome 12, one SNP significantly associated with IF was detected (Table 3 and Fig. 3). This SNP was approximately 10 Mb away from the QTL PVY‐12 previously detected for this trait on this chromosome (Fig. 5). The DH population used to detect PVY‐12 showed a lack of markers in the genomic region of the associated SNP (Tamisier *et al.*, 2017). Consequently, the mapping of PVY‐12 may not be precise enough and the associations detected with both methods could be caused by the same genomic region. Adding markers on the genetic map of the DH population could help to better interpret these results.

Two QTLs identified in a biparental population were not found with the association mapping approach: the VA‐3 QTL on chromosome 3 controlling VA (Quenouille *et al.*, 2014) and the PVY‐7 on chromosome 7 controlling *N*_e_ at inoculation (Tamisier *et al.*, 2017). A limit of GWAS is its lack of power to detect an association with a rare allele (Brachi *et al.*, 2011). We can assume that the frequencies of the resistance alleles at these two QTLs were too low in the core collection to be detected. It is probably the case for the VA‐3 QTL. Indeed, Quenouille *et al. *(2014) have shown that VA‐3 colocated tightly with the *pvr6* gene encoding eIFiso4E, an isoform of eIF4E encoded by the *pvr2* gene. In the biparental population, the *pvr* *6* ^+^ allele encodes a functional eIFiso4E protein, while the other *pvr6* allele is a ‘natural’ knockout (KO) of the gene, which encodes a truncated, non‐functional protein (Ruffel *et al.*, 2006). The pepper lines carrying the *pvr6* ^+^ allele displayed low VA while the lines carrying the KO allele displayed high VA. Within a subset of 20 pepper accessions of diverse origins, Quenouille *et al. *(2015) found that only three accessions carried the KO *pvr6* allele. If the KO *pvr6* allele frequency is still low at the core collection scale, it could explain why we did not detect this gene by association mapping. Given that the LD decays rapidly, another hypothesis would be that the marker density was not high enough to detect all the genomic regions controlling the traits.

Finally, the QTLs have been detected using two variants of the PVY‐SON41p isolate and we have not tested yet if their spectrum of action could be extended to other PVY strains. The PVY‐SON41p isolate belongs to the clade C1, which includes most of the PVY isolates infecting *Capsicum* spp. plants (Quenouille *et al.*, 2013b). This clade is quite diverse and we cannot generalize our results to all the isolates of the clade. However, at least two of the QTLs identified could have a broad spectrum of action. First, the SNP detected on chromosome 4 is most probably the *pvr2* resistance gene. This gene is already well‐known for providing a high level of resistance to diverse PVY and tobacco etch virus (TEV; genus *Potyvirus*, family *Potyviridae*) isolates (Moury *et al.*, 2004). Secondly, the SNP detected on chromosome 12 could be the QTL PVY‐12, which we have previously identified (Tamisier *et al.*, 2017). This QTL controls *N*_e_ during the inoculation step for both PVY and cucumber mosaic virus (CMV; genus *Cucumovirus*, family *Bromoviridae*), and therefore provides resistance against at least two viruses belonging to different families.

### Domestication favoured pyramiding of resistance QTLs to PVY‐SON41p variants in *C. annuum*

The distribution of the resistance alleles of the QTLs detected in this study among the *C. annuum* core collection has been analysed (Fig. S7). The resistance alleles of the QTLs on chromosome 4 were always associated with the resistance alleles of the QTLs on chromosomes 6, 9 or 12 more often than would be expected by chance. The resistance alleles of the QTLs on chromosomes 6 were also positively associated with those of the QTLs on chromosomes 9 or 12. Moreover, the LD between the markers of these QTLs ranged from 0.013 to 0.15. Although these values seem low, they were higher than the 55th, 60th, 70th, 80th, 85th or 90th percentiles of the interchromosomal LD distribution (Fig. S8), which demonstrates that the markers of these QTLs show higher LD than the background of the chromosomes, on average.

In the core collection, ten accessions belong to the wild subspecies *C. annuum* var. *glabriusculum*. The majority of these accessions carries a resistance allele at the QTLs on chromosomes 4 (seven to nine accessions, depending on the marker), 6 (nine to ten accessions) and 9 (nine accessions). None of these accessions carry a resistance allele at the QTL on chromosome 12, but few resistance alleles were present at this QTL in the core collection, the minor allele frequency of the SNP being low (2%). Even if the number of wild accessions in the core collection is small, our data tend to show that the resistance QTLs were already in association in the wild populations of *C. annuum*. As we have already seen, our results also demonstrate that the resistance QTLs are in coupling in the cultivated accessions. We can therefore hypothesize that these associations between resistance QTLs were advantageous for the plants, and that they have been partly maintained in pepper accessions through the process of crop domestication. Since the locus detected on chromosome 4 is most likely the major resistance gene *pvr2*, the domestication process could have selected for the pyramiding of multiple resistance QTLs, but also for the pyramiding of both resistance QTLs and major resistance genes. In past decades, multiple breeding strategies have been proposed to control plant disease (Zhan *et al.*, 2015). For instance, pyramiding resistance genes and resistance QTLs in the same cultivar is expected to protect major resistance genes from breakdown and to extend resistance durability (Brown, 2015; Palloix *et al.*, 2009). Our study demonstrates that pyramiding resistance loci has probably been occurring from early domestication until modern selection. The presence of pyramiding in pepper accessions coming from all over the world and that have been confronted with different PVY strains and potyvirus species is more evidence that this combination could be a promising breeding strategy to achieve durable resistance.

## Experimental Procedures

### Pepper core collection sampling

The pepper germplasm (*Capsicum* spp.) maintained in the Institut National de la Recherche Agronomique (INRA) in Avignon is composed of 1352 non‐redundant accessions, including 11 cultivated and wild species, which originated from 89 countries and five continents (Sage‐Palloix *et al.*, 2007). The pepper collection is mostly composed of *C. annuum* inbred lines (78.6%) that can be divided into the cultivated subspecies *C. annuum* var. *annuum*, which represents more than 90% of the *C. annuum* accessions, and the wild subspecies *C. annuum* var. *glabriusculum*. All the accessions have been previously genotyped with a set of 28 simple sequence repeat (SSR) markers (Nicolaï *et al.*, 2012). They have also been tested for their resistance to three PVY isolates, which differ by their capacity to infect plants carrying different alleles at the *pvr2* resistance gene. One isolate belonged to pathotype PVY‐0 and therefore infects only accessions carrying the susceptibility allele *pvr2* ^+^. The second isolate belonged to pathotype PVY‐0,1 and infects plants carrying the *pvr2* ^+^or *pvr2* ^1^ alleles, and the third one from pathotype PVY‐0,1,2,3 infects plants carrying either the *pvr2* ^+^, *pvr2* ^1^, *pvr2* ^2^ or *pvr2* ^3^ alleles.

The viral clones used to phenotype the core collection belonged to pathotype PVY‐0,1,3. Therefore, from the whole collection, we excluded all the *C. annuum* accessions resistant to pathotype PVY‐0,1,2,3. This choice ensured that the majority of the selected accessions would be infected during phenotyping and that we could measure quantitative resistance. The core collection of *C. annuum* was built using MStrat software v. 4.1 (Gouesnard *et al.*, 2001) on the SSR dataset of Nicolaï *et al. *(2012). We first estimated the minimum number of accessions needed to keep the same allelic richness as the whole germplasm. To do this, we performed sampling simulations for different sizes of core collection with the maximization (M) strategy algorithm and calculated the increase in allelic richness for all these core collections. The plateau of this curve indicates the minimum number of accessions displaying the same genetic diversity as the germplasm (Fig. S1). We then applied the M strategy to select the final core collection, with 20 replicates and 30 iterations per replicate. The most prevalent accessions among the 20 replicates were included in the final core collection. The number of alleles, the Nei’s unbiased gene diversity index (*H*_e_) and the observed heterozygosity (*H*_o_) of the core collection were calculated using GenAlEx v. 6.5 software (Peakall and Smouse, 2012).

### Phenotyping of the core collection

Two traits were phenotyped among the core collection: the number of PVY primary infection foci (IF) and the relative PVY accumulation (VA). Two viruses deriving from a cDNA clone of PVY isolate SON41p and belonging to the pathotype PVY‐0,1,3 were used. Both clones carried a substitution in the VPg, the 115K or the 119N, which allows them to overcome the *pvr2* ^3^ resistance. In order to obtain VA values that best discriminate between the accessions, the 119N clone was used because it overcomes the resistance gene with an average efficiency, whereas the 115K clone shows on average a high fitness (Rousseau *et al.*, 2018). To measure IF, a clone expressing the GFP marker was needed. Since we expected the GFP reporter gene to decrease the fitness of the virus, the 115K clone tagged with the GFP was used to counterbalance this fitness loss.

The core collection was sown twice with 20 and 10 plants per accession for IF and VA, respectively. Both viruses were first propagated in *Nicotiana tabacum* 'Xanthi' plants. Then, the two cotyledons of each plant were mechanically inoculated 28 days after sowing, for both PVY clones. For IF, the number of foci showing green fluorescence was quantified 5 days post‐inoculation under specific light wavelength (450–490 nm) for 20 cotyledons per accession. For VA, 1 g from three uninoculated apical leaves was sampled from each inoculated plant 1 month post‐inoculation. Samples from each plant were separately ground in a phosphate buffer (0.03 M Na_2_HPO_4_, 0.2% sodium diethyldithiocarbamate, 4 mL buffer/gram of leaves). To reduce the number of samples and make the VA estimation achievable, the ten samples of ground leaves per accession were pooled into two groups of four samples and one group of two samples, reducing the experiment from ten measures of VA per accession to three. A control experiment had been performed previously on 30 plants to confirm that the estimation of VA was accurate when using pooled samples (Fig. S9). A quantitative double antibody sandwich–enzyme‐linked immunosorbent assay (DAS‐ELISA) was performed on each pooled sample as described by Ayme *et al. *(2006). The mean virus concentration per accession was assessed using serial dilutions of the pooled samples of infected plants and calculated relative to a common control sample added to each ELISA plate. Finally, a relative virus concentration was obtained for each accession.

### Sequencing strategy for SNP detection

The core collection was sown a third time in greenhouse conditions. One plant per accession was used for DNA extraction. DNA was isolated from 80 mg of frozen young leaves with the DNeasy Plant Mini Kit (Qiagen, Marseille, France) and the extracted DNA was quantified using a Qubit fluorometer (Thermo Fisher Scientific, Illkirch, France). DNA of high quality was obtained for 276 accessions, which were used to perform GBS. The genome complexity was reduced by ddRADseq (Peterson *et al.*, 2012). The two restriction enzymes used were *Pst*I, a rare‐cutting restriction enzyme sensitive to methylation, and *Mse*I, a common‐cutting restriction enzyme. Adapters were then ligated to restriction fragments, the samples were pooled and PCR amplifications were performed. All the libraries were constructed at the Cirad facilities (Montpellier, France). Next‐generation sequencing was performed in a 150‐bp single‐read mode using three lanes on a HiSeq3000 platform (Illumina, San Diego, CA, USA) at Genotoul (Toulouse, France).

The FASTQ files were demultiplexed using a python script that searches for the adapters in 5ʹ ends and for the expected restriction sites following the adapters. It also removes the adapter sequences in the 5ʹ ends (https://github.com/timflutre/quantgen/blob/master/demultiplex.py). Adapters were then removed in the 3ʹ ends and the sequence reads were filtered for quality (quality trimming > 20 and minimum read length = 35 bp) using cutadapt (Martin, 2011). The reads were aligned against the reference genome of *C. annuum* cv. CM334 version 1.55 (Kim *et al.*, 2014) using the Burrows–Wheeler aligner tool and the algorithm BWA‐MEM (Li, 2013). Variant calling was performed using Genome Analysis Toolkit (GATK) haplotypecaller (McKenna *et al.*, 2010). A raw HapMap file was produced with TASSEL v. 5.2.39 (Bradbury *et al.*, 2007) and several in‐house R scripts were used to filter SNPs. Accessions showing more than 30% of missing data were discarded. A minor allele frequency (MAF) of 2% was set according to the formula of Aulchenko *et al. *(2007). Imputation of missing SNPs was performed with the Random Forest Regression Imputation Model implemented in the R package ‘missForest’ (Stekhoven and Bühlmann, 2011).

### Population structure and linkage disequilibrium estimations

Population structure was estimated using the Bayesian model‐based clustering method implemented in STRUCTURE v. 2.3.4 software (Pritchard *et al.*, 2000). The admixture model was used with 100 000 replicates for burn‐in and Markov chain Monte Carlo (MCMC) iteration. Five runs were performed for each value of clusters (*K*), ranging from 1 to 10. The optimal *K* value was inferred from the log probability of the data (Ln*P*(D)) and delta *K* (Evanno *et al.*, 2005). These values were calculated with Structure Harvester (Earl, 2012). A neighbour‐joining tree based on pairwise genetic distances between SNPs was constructed using DARwin v. 6.0 software (Perrier and Jacquemoud‐Collet, 2006). Linkage disequilibrium (LD) analysis was performed with the LDcorSV package implemented in R (Mangin *et al.*, 2012), using the LD squared allele frequency correlation (*r* ^2^) estimated from pairwise comparisons between SNPs.

### Genome‐wide association study

The GWAS analyses were performed using two models in order to compare the SNPs detected and obtain more robust results. First, we used the CMLM (Zhang *et al.*, 2010) implemented in the GAPIT R package (Lipka *et al.*, 2012). The CMLM accounts for the structure of the population by including principal components as fixed effects and for relatedness between accessions by including a random‐effect kinship matrix (K matrix), where kinship estimates are calculated between pairs of groups. The FDR correction was used to account for multiple testing and determine a corrected significance cut‐off. We also used the multilocus mixed‐model (MLMM) developed by Segura *et al. *(2012). The MLMM is based on a stepwise mixed‐model regression with forward inclusion and backward elimination. At each step of the regression, genetic and error variances are re‐estimated, therefore the MLMM allows the use of multiple cofactors in the model, as done in traditional linkage mapping methods, and takes into account better the confounding effects of background loci (i.e. linkage disequilibrium). The best model has been chosen using the multiple‐Bonferroni criterion (mBonf).

### Statistical analyses

Statistical analyses were performed using R software (http://www.r-project.org/). For the two phenotypic traits (IF and VA), broad‐sense heritability was estimated using the formula *h* ^2^ = *σ* ^2^_G_ /(*σ* ^2^_G_ + *σ* ^2^_E_/*n*), where *σ* ^2^_G_ corresponds to the genotypic variance, *σ* ^2^_E_ to the environment variance and *n* to the number of replicates (*n* = 20 and 3, respectively). A log(*x* + 1) transformation was applied to the number of PVY primary IF and a cube‐root transformation was applied to the VA to approximate a normal distribution. Associations between susceptibility and resistance alleles for pairwise SNPs among the core collection were analysed using chi‐squared tests and Monte Carlo simulations (10 000 000 replicates).

## Funding Information

L. Tamisier’s PhD was supported by the Biologie et Amélioration des Plantes department and Sustainable Management of Crop Health (SMaCH) metaprogramme of INRA and by the Région Provence‐Alpes‐Côte d’Azur. The experimental work was supported by the SMaCH metaprogramme and the ANR PRC project ArchiV (2019‐2022).

## Conflicts of Interest

The authors declare that there are no conflicts of interest.

## Supporting information


**Fig. S1** Allelic richness (score) capture for different core collection sizes. A random (red) and a maximization (black) sampling strategies are represented.

**Fig. S2** Polymorphisms distribution in the 12 pepper chromosomes for (a) all SNPs, (b) insertion polymorphisms and (c) deletion polymorphisms. The number of SNP, insertion or deletion is indicated in brackets for each chromosome.

**Fig. S3** Linkage disequilibrium decay (*r* ^2^) against the genetic distance (bp) of each chromosome throughout the CM334 pepper reference genome.

**Fig. S4** Delta *K* values calculated by Evanno’s method.

**Fig. S5** Morphological and developmental phenotypes of the accessions in the *Capsicum annuum *core collection. For each plot, the accessions are distributed among the four clusters determined by the structure analysis. The whole INRA pepper germplasm (*Capsicum* spp.) has been previously characterized for several plant and fruit traits (Sage‐Palloix *et al.*, 2007). The traits are (a) the fruit length (cm), (b) the fruit width (cm), (c) the fruit weight (gram), (d) the pericarp thickness (mm), (e) the flowering earliness, which represents the number of days between sowing and first anthesis and which is expressed relatively to a control genotype (called Yolo Wonder) and (f) the number of leaves on the primary axis. The letters a and b indicate the different groups obtained after pairwise comparisons using the Nemenyi test (*P* < 0.05).

**Fig. S6** Distribution of pairwise kinship estimates across the core collection.

**Fig. S7** Distribution of the pepper accessions according to the alleles they carry for six pairs of SNPs belonging to different chromosomes. The two bars split the accessions according to the allele at the first SNP, and the black and grey colours split the accessions according to the allele at the second SNP. Chi‐squared test with Monte Carlo simulation (100 000 000 replicates) was used to compare accession distribution among the different categories (*P < 0.05, **P < 0.01, ***P < 0.001).

**Fig. S8** Distribution of interchromosomal linkage disequilibrium (LD) between (a) chromosomes 4 and 6, (b) chromosomes 4 and 9, (c) chromosomes 4 and 12, (d) chromosomes 6 and 9, and (e) chromosomes 6 and 12. LD between pairs of SNPs detected in genome‐wide association studies are indicated by dotted lines.

**Fig. S9** Control experiment for the accuracy of the virus accumulation measured with pooled samples. Two pepper accessions with 15 plants per accession were inoculated with a cDNA clone of PVY isolate SON41p. Apical leaves of each plant were ground in a phosphate buffer (0.03 M Na_2_HP0_4_, 0.2% sodium diethyldithiocarbamate, 4 mL buffer/gram of leaves) and the virus accumulation was assessed with a quantitative DAS‐ELISA for each plant. Samples of ground leaves were then pooled. For each accession, 1.8 mL of ground leaves belonging to two different plants and 0.9 mL of ground leaves belonging to four different plants were pooled together. Once again, a DAS‐ELISA was performed to quantify the virus accumulation of the pooled samples. The relative virus accumulation obtained for the individual plants and the pool of (a) two and (b) four plants was compared for the two pepper accessions (illustrated by circles and triangles). Significant positive correlations were found for both types of pools.

**Table S1** List of the 256 *Capsicum* *annuum* accessions of the core collection and clustering of these accessions based on population structure. For 50 accessions, the alleles at the locus *pvr2* are given.

**Table S2** SNPs identified with genome‐wide association studies and associated with the number of primary infection foci induced by PVY‐GFP (IF) and PVY accumulation (VA) in pepper when excluding the wild subspecies *C. annuum *var.* glabriusculum* from the core collection. NS: not significant.

**Table S3** Number of accessions carrying a resistance (R) or susceptibility (S) allele at the most significant SNPs identified on chromosomes 4 (positions 1 151 249 and 340 333 bp), 6 (position 234 143 013 bp), 9 (position 58 056 303 bp) and 12 (position 235 513 719 bp). For the two SNPs analysed at chromosome 4, when at least one of them was carrying a resistance allele, the accession was considered to carry a resistance allele at this chromosome.

**Table S4** Candidate genes associated with the SNPs detected for the number of primary infection foci and the virus accumulation.

**Text S1 **Candidate genes in QTL confidence intervals that could be linked to PVY resistance.
