# Supplementary figures and images for: Genome‐wide association mapping of QTLs implied in potato virus Y population sizes in pepper: evidence for widespread resistance QTL pyramiding
Source: Mol Plant Pathol. 2019 Oct 11;21(1):3–16. doi: 10.1111/mpp.12874 (PMC6913244; doi:10.1111/mpp.12874)

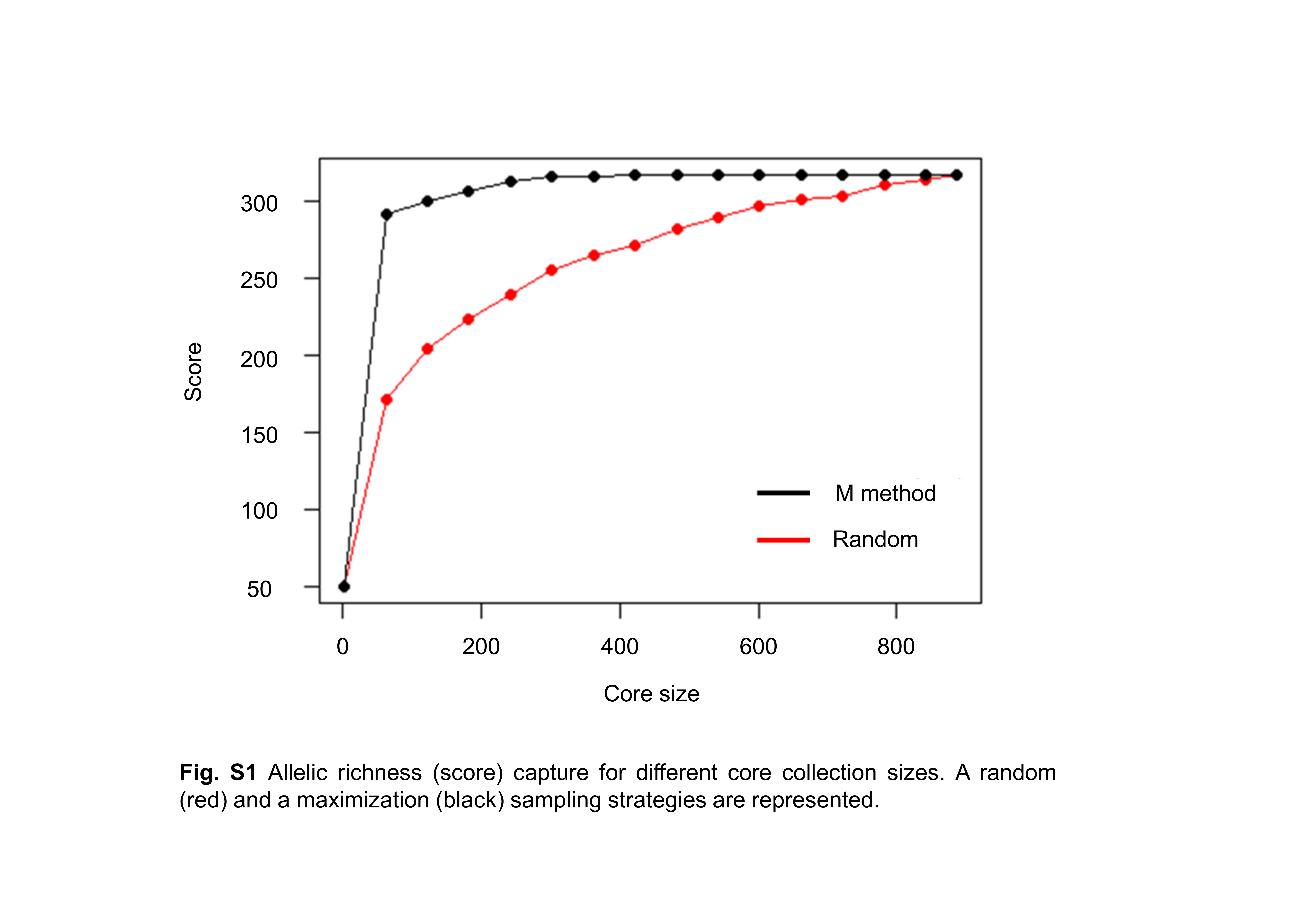

Supplement: Supplementary file 1 — Fig. S1 Allelic richness (score) capture for different core collection sizes. A random (red) and a maximization (black) sampling strategies are represented. [file MPP-21-3-s001.tif]

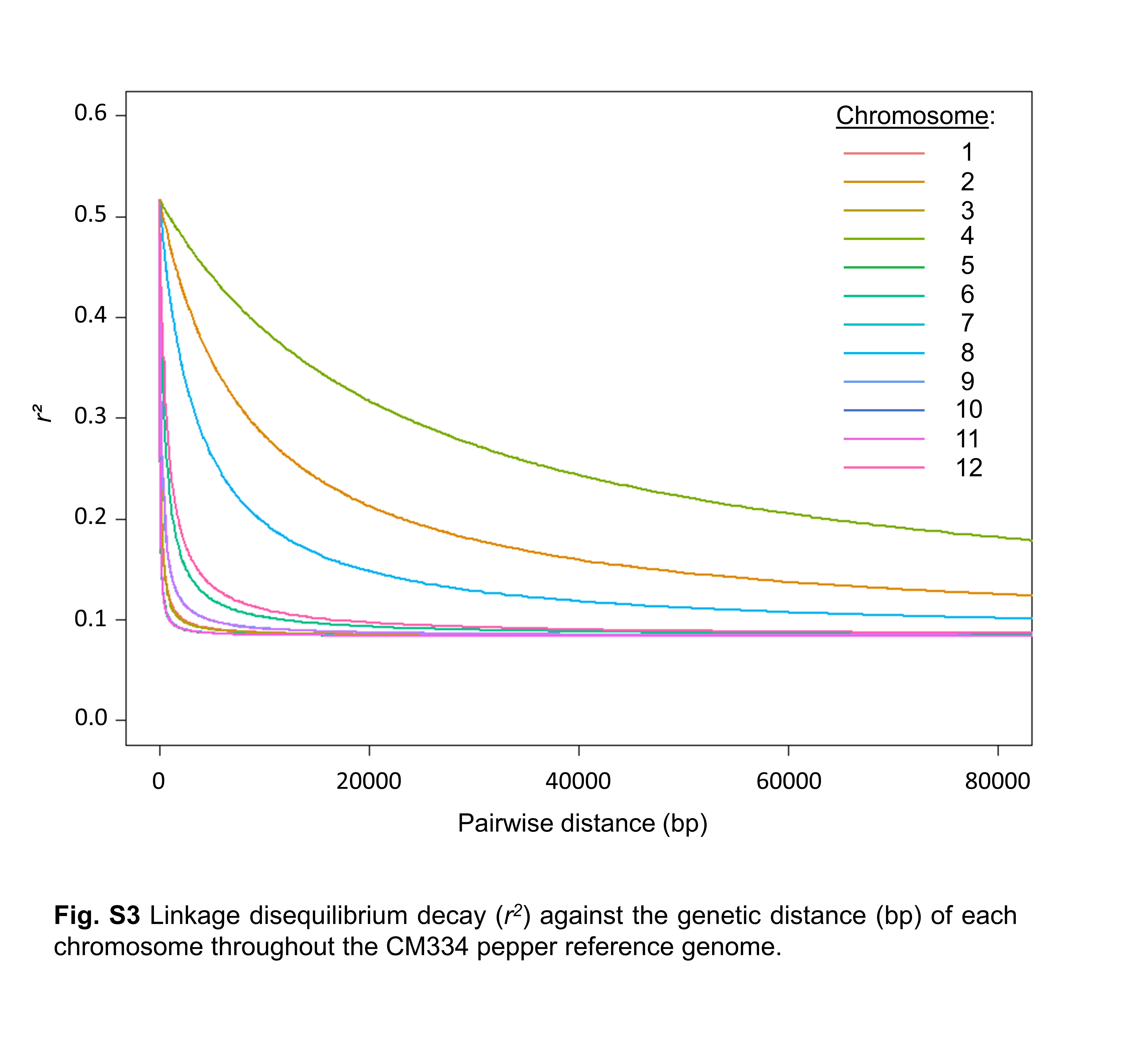

Supplement: Supplementary file 3 — Fig. S3 Linkage disequilibrium decay (r 2) against the genetic distance (bp) of each chromosome throughout the CM334 pepper reference genome. [file MPP-21-3-s003.tif]

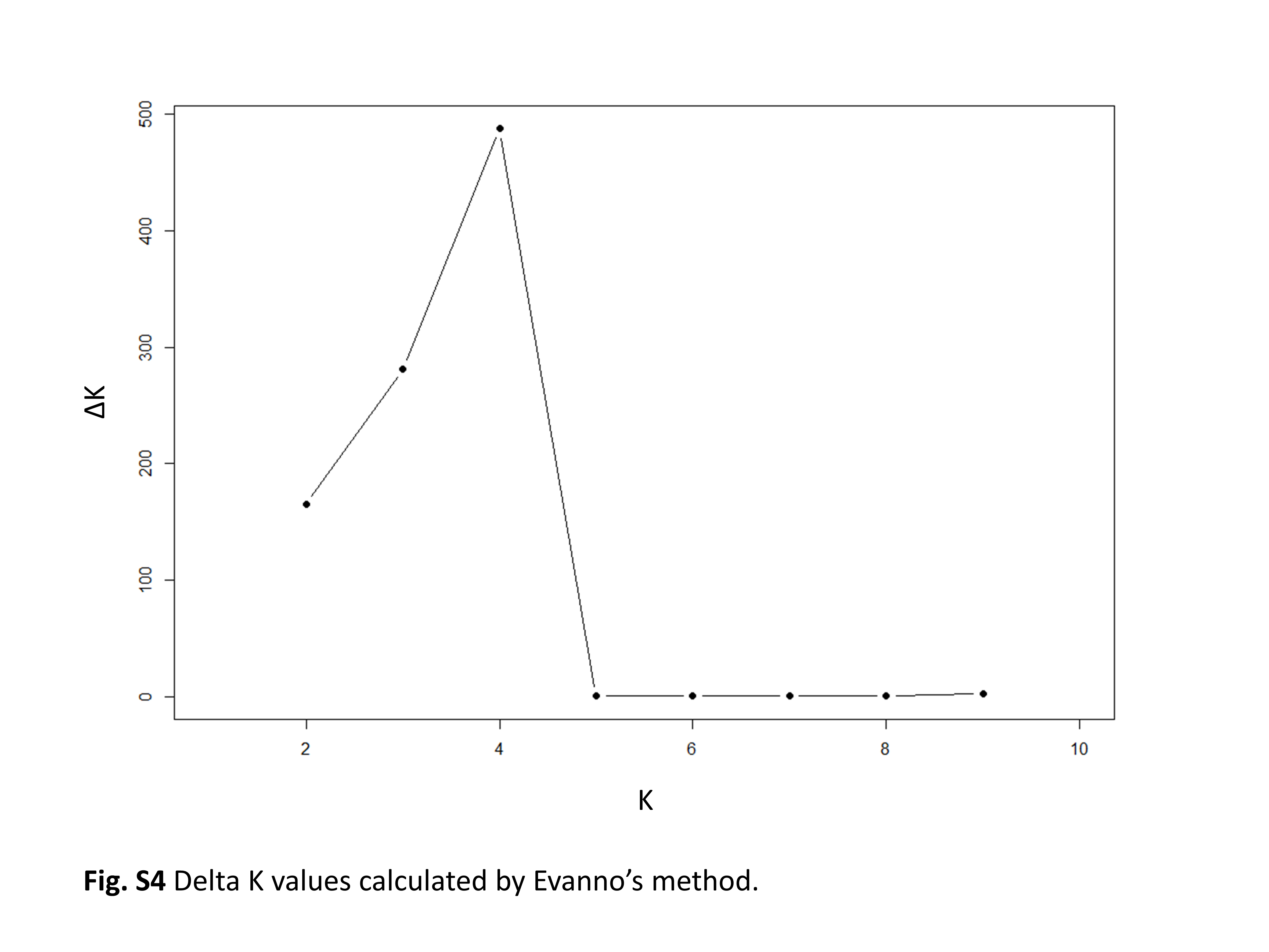

Supplement: Supplementary file 4 — Fig. S4 Delta K values calculated by Evanno’s method. [file MPP-21-3-s004.tif]

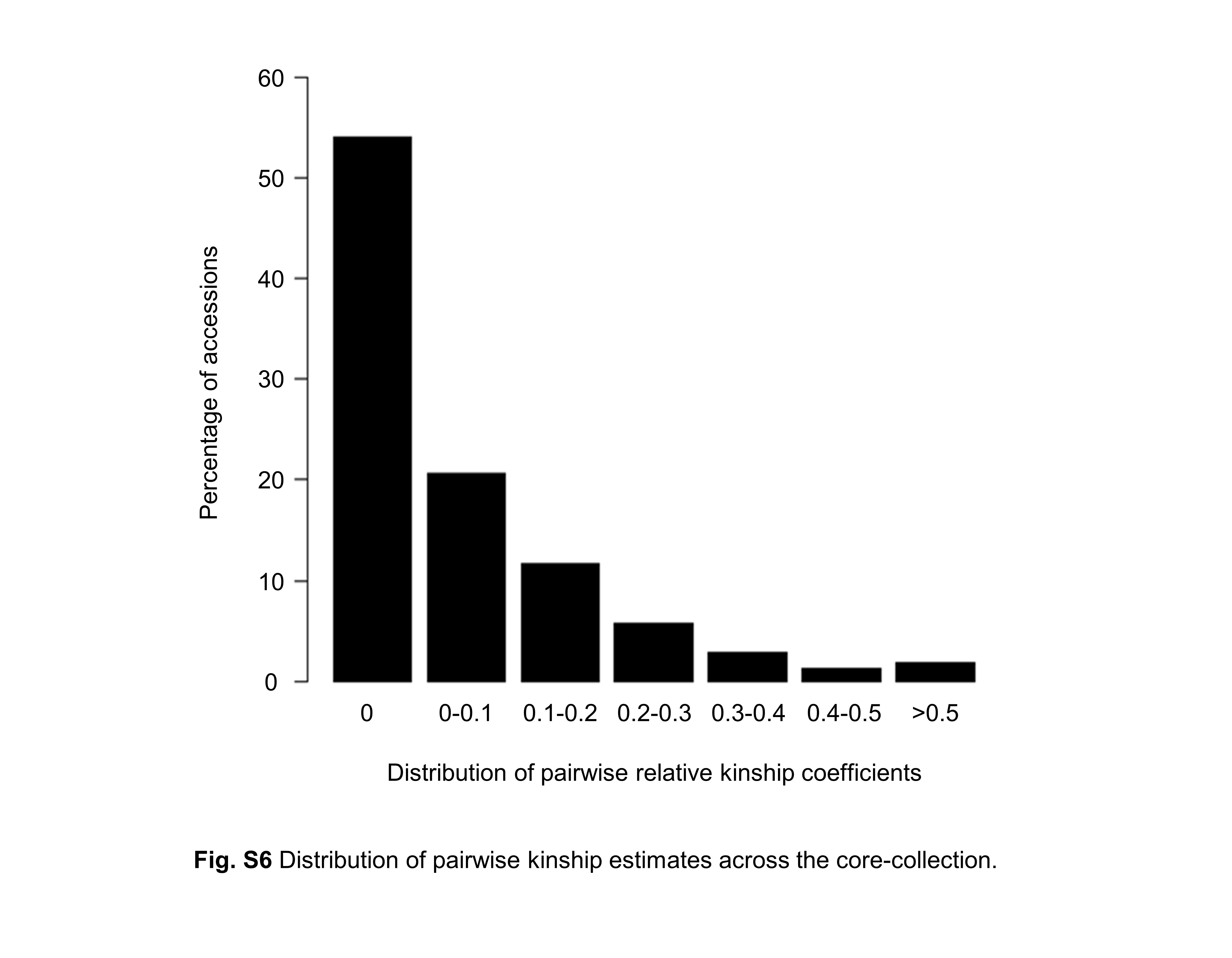

Supplement: Supplementary file 6 — Fig. S6 Distribution of pairwise kinship estimates across the core collection. [file MPP-21-3-s006.tif]

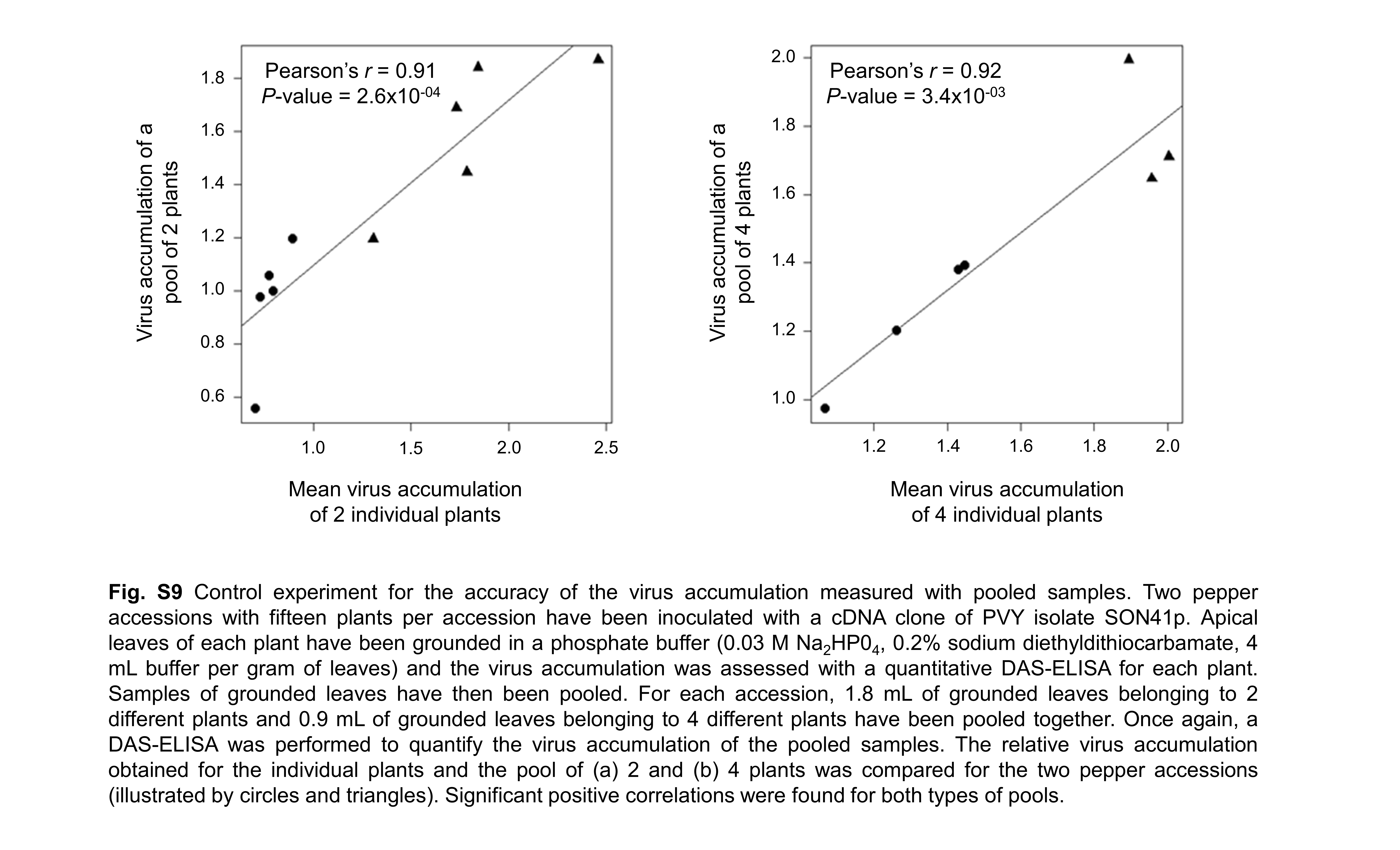

Supplement: Supplementary file 9 — Fig. S9 Control experiment for the accuracy of the virus accumulation measured with pooled samples. Two pepper accessions with 15 plants per accession were inoculated with a cDNA clone of PVY isolate SON41p. Apical leaves of each plant were ground in a phosphate buffer (0.03 M Na2HP04, 0.2% sodium diethyldithiocarbamate, 4 mL buffer/gram of leaves) and the virus accumulation was assessed with a quantitative DAS‐ELISA for each plant. Samples of ground leaves were then pooled. For each accession, 1.8 mL of ground leaves belonging to two different plants and 0.9 mL of ground leaves belonging to four different plants were pooled together. Once again, a DAS‐ELISA was performed to quantify the virus accumulation of the pooled samples. The relative virus accumulation obtained for the individual plants and the pool of (a) two and (b) four plants was compared for the two pepper accessions (illustrated by circles and triangles). Significant positive correlations were found for both types of pools. [file MPP-21-3-s009.tif]
