# Supplementary material for: Genome‐wide association mapping of QTLs implied in potato virus Y population sizes in pepper: evidence for widespread resistance QTL pyramiding
Source: Mol Plant Pathol. 2019 Oct 11;21(1):3–16. doi: 10.1111/mpp.12874 (PMC6913244; doi:10.1111/mpp.12874)
Supplement: Supplementary file 14 — Text S1 Candidate genes in QTL confidence intervals that could be linked to PVY resistance. [file MPP-21-3-s014.docx]

**Text S1: Candidate genes in QTL confidence intervals that could be linked to PVY resistance**

In this study, we performed genome-wide association studies (GWAS) on a *Capsicum annuum* core-collection. We identified 101 putative candidate genes that could be involved in the number of *Potato virus Y* (PVY) infection foci at inoculation and in the virus accumulation at the systemic level (Table S4).

On chromosome 4, we found 19 candidate genes that could affect both traits. The best candidate is the major resistance gene *pvr2*, which encodes an eIF4E (eukaryotic initiation factor 4E). First, this gene is well-known for conferring recessive resistance against some strains of PVY. Then, the single nucleotide polymorphisms (SNPs) detected for both traits on chromosome 4 are the closest markers of the *pvr2* gene among all SNPs (Figure 7). To confirm that *pvr2* could be responsible for PVY resistance differences in the pepper core-collection, we sequenced the corresponding open reading frame of 50 accessions randomly chosen among the resistant or susceptible phenotypic classes (Table S1). The *pvr2* open reading frame from the 50 pepper accessions was amplified by RT-PCR using specific primers as described in Ben Khalifa *et al.* (2009), and the resulting PCR products were directly sequenced by Genoscreen (Lille, France). All accessions but one were homozygous at the *pvr2* allele. The most frequent *pvr2* alleles were the susceptibility allele *pvr2+* (20 cases) and the resistance alleles *pvr2^1^* (10 cases) and *pvr2^3^* (7 cases). Alleles *pvr2^2^*, *pvr2^4^*, *pvr2^23^* and two new alleles (corresponding either to the combination of amino acid substitutions V67E and A73D or to the amino acid substitution K71R in the eIF4E) were observed at low frequencies (≤ 4 cases). In this accession subset, the mean IF of the accessions carrying the susceptibility allele (*pvr2+*) was compared to the mean IF of the accessions carrying the resistance alleles (all other *pvr2* alleles) using the Wilcoxon sum rank test. The difference between both groups was highly significant (p = 0.006). The difference was lower or not significant when comparing the mean IF of the accessions carrying the resistance or the susceptibility allele at each SNP of chromosome 4 (0.057 ≥ p ≥ 0.019), confirming the probable involvement of the *pvr2* gene. For VA, the comparisons between groups of *pvr2* or SNP alleles did not show any significant differences, probably because the number of accessions was too low to reveal differences for this variable. However, the p-value was still lower for the *pvr2* (p = 0.198) than for the SNP (0.981 ≥ p ≥ 0.632) alleles.

Detecting significant resistance differences among accessions different at the *pvr2* locus is unexpected since (i) the majority of accessions showing the highest resistance (lowest IF and/or VA) carried the *pvr2^1^* and *pvr2^3^* alleles and (ii) the PVY clones used for phenotyping belonged to pathotype PVY-0,1,3 and hence had the capacity to overcome the resistances conferred by *pvr2^1^* and *pvr2^3^*. One hypothesis to explain this result is that several of the *pvr2* alleles, including at least *pvr2^1^* and *pvr2^3^*, have “residual” quantitative resistance effects against PVY populations carrying resistance-breaking mutations.

Other good candidate genes have also been identified on chromosome 4. For instance, a leucine-rich repeat (LRR) receptor-like serine threonine kinases and a putative LRR receptor belong to the confidence interval around the associated SNPs. The LRR domains are known to be involved in pathogens recognition and plant defense (Afzal *et al.*, 2008), and they could therefore be implied in PVY resistance. A putative phosphatidylinositol 4-kinase has also been identified. Recently, Feng *et al.* (2019) have demonstrated that another member of the phosphoinositide family, the Vps34 phosphatidylinositol 3-kinase, is required for the formation of the viral replication compartment within the *Tombusviridae* family, both in yeast and plant cells. We can therefore hypothesize that the phosphatidylinositol 4-kinase could also play a role during PVY infection.

On chromosome 6, 32 candidate genes that could affect both traits have been found. One of them encodes a cytochrome CYP82M1v4. In tobacco, Takemoto *et al.* (1999) have shown that the cytochrome CYP82E1 may be involved in resistance against *Phytophthora infestans* and *Pseudomonas syringae* infections. Moreover, in pepper, the cytochrome P450 could play a role in the defense mechanisms against the anthracnose fungus *Colletotrichum gloeosporioides* (Oh *et al.*, 1999). The cytochrome CYP82M1v4 could therefore be a good candidate to be investigated for its potential role in resistance against viruses. Even if the other candidates are not directly correlated to plant defense against pathogens, we can still make assumptions regarding their potential function. For example, three genes coding for a wound-inducible carboxypeptidase have been reported. This protein accumulate in tomato leaves in response to wounding or methyl jasmonate treatment (Dı́ez-Dı́az *et al.*, 2004), and could therefore play a role in the plant defense system.

On chromosome 9, 25 candidate genes potentially involved in the number of PVY infection foci at inoculation have been identified. Among them, a vacuolar sorting-associated protein YPR157W has been identified. This protein is required for vesicle trafficking towards the vacuole. Recently, Giner *et al.* (2017) have shown that a vacuolar protein sorting 41 avoids the systemic infection of *Cucumber mosaic virus* (CMV) in melon, notably by impeding viral loading into the phloem. Since the QTL that we have detected reduces PVY population sizes at inoculation, this gene could be a good candidate. None of the others candidate genes on this chromosome are directly linked to plant defense against pathogens. However, one of them encodes a putative auxin-responsive protein. Auxin being a phytohormone affecting plant defense (Wang and Fu, 2011), it could be implied in plant response against PVY.

On chromosome 12, 25 candidate genes that could affect the number of PVY infection foci at inoculation have been identified. Among them, a pleiotropic drug resistance protein 1-like has been reported. Most of these proteins are known to be involved in plant general defense mechanisms, in response to either abiotic or biotic stress (Crouzet *et al.*, 2006). A protein similar to the phosphatase 2A-associated protein Tap46 has also been found. Ahn *et al.* (2011) have highlighted that Tap46 plays an essential role in the target of rapamycin (TOR) signaling pathway, which regulates cellular metabolic status and growth in most eukaryotes. Moreover, the inhibition of TOR activity in *Arabidopsis* has been found to strongly decrease *Watermelon mosaic virus* (WMV) accumulation, while it only caused a delay in infection for plants infected with *Turnip mosaic virus* (TuMV). Since the TOR signaling pathway is needed for the establishment and maintenance of some potyvirus species, this pathway, and to a larger extent the Tap46 protein, could also be involved in PVY infection process. Finally, the closest candidate gene to the SNP detected on this chromosome is supposed to encode for an E3 ubiquitin ligase (SINAT2). This protein is known to mediate ubiquitination and promoting degradation of target proteins (Qi *et al.*, 2017). In the ubiquitin/26S proteasome system, the E3 ubiquitin ligase is involved in the defence of plants against pathogens (Dielen *et al.*, 2010), including early defence reactions, gene-for-gene interactions and induced disease resistance (Delaure *et al.*, 2008; Zeng *et al.*, 2006).

**References:**

**Afzal, A.J., Wood, A.J. and Lightfoot, D.A.** (2008) Plant receptor-like serine threonine kinases: roles in signaling and plant defense. *Mol. Plant. Microbe Interact.* **21**, 507–517.

**Ahn, C.S., Han, J.-A., Lee, H.-S., Lee, S. and Pai, H.-S.** (2011) The PP2A regulatory subunit Tap46, a component of the TOR signaling pathway, modulates growth and metabolism in plants. *Plant Cell*, tpc-110.

**Ben Khalifa, M., Simon, V., Marrakchi, M., Fakhfakh, H. and Moury, B.** (2009) Contribution of host plant resistance and geographic distance to the structure of Potato virus Y (PVY) populations in pepper in northern Tunisia. *Plant Pathol.* **58**, 763–772.

**Crouzet, J., Trombik, T., Fraysse, Å.S. and Boutry, M.** (2006) Organization and function of the plant pleiotropic drug resistance ABC transporter family. *Febs Lett.* **580**, 1123–1130.

**Delaure, S.L., Van Hemelrijck, W., De Bolle, M.F., Cammue, B.P. and De Coninck, B.M.** (2008) Building up plant defenses by breaking down proteins. *Plant Sci.* **174**, 375–385.

**Dielen, A., Badaoui, S., Candresse, T. and German‐Retana, S.** (2010) The ubiquitin/26S proteasome system in plant–pathogen interactions: a never‐ending hide‐and‐seek game. *Mol. Plant Pathol.* **11**, 293–308.

**Dı́ez-Dı́az, M., Conejero, V., Rodrigo, I., Pearce, G. and Ryan, C.A.** (2004) Isolation and characterization of wound-inducible carboxypeptidase inhibitor from tomato leaves. *Phytochemistry* **65**, 1919–1924.

**Feng, Z., Xu, K., Kovalev, N. and Nagy, P.D.** (2019) Recruitment of Vps34 PI3K and enrichment of PI3P phosphoinositide in the viral replication compartment is crucial for replication of a positive-strand RNA virus. *PLoS Pathog.* **15**, e1007530.

**Giner, A., Pascual, L., Bourgeois, M., et al.** (2017) A mutation in the melon Vacuolar Protein Sorting 41prevents systemic infection of Cucumber mosaic virus. *Sci. Rep.* **7**, 10471.

**Oh, B.-J., Ko, M.K., Kim, Y.S., Kim, K.S., Kostenyuk, I. and Kee, H.K.** (1999) A cytochrome P450 gene is differentially expressed in compatible and incompatible interactions between pepper (Capsicum annuum) and the anthracnose fungus, Colletotrichum gloeosporioides. *Mol. Plant. Microbe Interact.* **12**, 1044–1052.

**Qi, H., Xia, F.-N., Xie, L.-J., et al.** (2017) TRAF-Family Proteins Regulate Autophagy Dynamics by Modulating AUTOPHAGY PROTEIN6 Stability in Arabidopsis. *Plant Cell*, tpc-00056.

**Takemoto, D., Hayashi, M., Doke, N., Nishimura, M. and Kawakita, K.** (1999) Molecular cloning of a defense-response-related cytochrome P450 gene from tobacco. *Plant Cell Physiol.* **40**, 1232–1242.

**Wang, S. and Fu, J.** (2011) Insights into auxin signaling in plant–pathogen interactions. *Front. Plant Sci.* **2**, 74.

**Zeng, L.-R., Vega-Sánchez, M.E., Zhu, T. and Wang, G.-L.** (2006) Ubiquitination-mediated protein degradation and modification: an emerging theme in plant-microbe interactions. *Cell Res.* **16**, 413.
